# Abstracts

**Published:** 1882-07

**Authors:** 


### Abstracts.

*Effects of Malaria on Man.*—In addition to the direct effects of malaria, a diathetic condition seems to be established which modifies other diseases. The effects of malaria are, indeed, most protean in form, not only in its own definite and well-marked pathological processes, but it simulates others. The stupor of typhus, the collapse of cholera, the high temperature of insolation, the sickness of an irritant poison, the convulsions of epilepsy or of dentition, may occur in the pernicious forms. It induces anæmia and general cachexia, with structural changes in the liver, spleen, or other viscera, neuralgia, asthma, and various other symptoms of disturbed innervation and sanguification, and it also appears to be in close etiological relation with dysentery, cholera, diarrhœa, beriberi, hydrocele, elephantiasis, bronchocele and hepatic disease. Whatever its nature may be, its action on the human economy is very striking; it affects the central nervous system, causing disturbance of vaso-motor action, paroxysms of fever, and congestion of the abdominal viscera, which may become periodic in recurrence, or pass on to structural changes in the liver and spleen, or intestinal mucous membrane. No one can have resided long in a malarious climate, such as Assam, without observing the cachectic, deteriorated aspect of the people, who, although they may never have had a single attack of fever, scarcely feel ill, and would resent being told so, are yet victims to the insidious action of the poison, and present evidences of anæmia, degenerate tissues and chronic visceral disease.—Croonian Lectures. Sir Joseph Fayrer, M.D., F.R.S., etc.

*Treatment of Epidemic Cerebro-Spinal Meningitis.*—We cull from the *Medical Times* the treatment advocated by Dr. Karl

Jaffé: The therapeutics of this disease are quickly summed up. The author approves of the advice of the older observers with regard to the ice treatment applied to the spine, and the use of narcotics, without which we can scarcely succeed even in the lightest cases. Blood-letting has only a very transitory effect. Of remedies, laxatives, especially calomel in large doses, are especially valuable. Mercurial inunctions in one case were without any influence worth mentioning. Prolonged bathing cannot generally be carried out, yet tepid and cool baths, when the temperature is high, are very useful. The medicamental antipyretics (quinia, salicylic acid, and also sodium benzoate) may be entirely discarded, because they are more uncertain than the baths, and they ruin the already weakened digestion. He found that the sick were always worse after their use, in the cases in which they were tried, so he finally gave it up. This medical nihilism will appear justified if we consider how largely those affected with meningitis perish, especially with the rapid form of the disease, how protracted is the convalescence, and what lasting injury must follow the use of every remedy and every means (we might here also include blood-letting), which in any way alters the digestive tract and affects the tissue-changes of the patient.

*Icterus Neonatorum.*—Birch Hirschfield, in an article in Virchow's *Archiv*, an abstract of which appears in the London *Lancet*, gives the following as the pathology of that hitherto obscure disease: It is very difficult to avoid associating the jaundice in some way with the disturbance of the hepatic circulation on the transfer of its chief blood supply from the umbilical vein, especially when regard is had to the conspicuous congestion and œdema of the liver, well described by Weber, which occur in cases in which the circulation through the umbilical cord is interrupted before the respiratory movements, by their effect on the right heart, afford an adequate compensation. The vessels in the hilus of the liver are surrounded by a dense layer of connective tissue, which is continued into the organ along the branches of the portal vein, and in cases in which there is venous obstruction in the liver, in consequence of hindered birth, this tissue is the seat of conspicuous œdema. A broad layer of gray pulpy tissue encloses the vessels, and is seen also around the umbilical vein in its diaphragmatic portion, and may also extend to the gall-bladder. The microscopical appearances of this tissue are those of œdema, with a more or less abundant accumulation of round cells in the interstices of the tissue. This swelling of the tissue must compress the bile-ducts obviously, and, not only under these circumstances are the bile-ducts distended, but there may be a positive difficulty in squeezing the bile out of the gall-bladder in the duodenum, and in the latter there is a manifest deficiency of bile. In such cases, in which death occurs during the first day of life, commencing icterus may be distinctly detected, and the gradual increase of the jaundice in connection with this pathological condition, may be observed in patients in whom life continues longer, as cases reported by Birch Hirschfeld demonstrate.

*Cholera in West Africa.*—Recent information brings definite news of the imposition of quarantine, both maritime and inland, at British stations on the Gold Coast on account of cholera prevailing more or less among the Mohammedan population on the Upper Niger. The outbreak is believed to be an offshoot of the prevalence of the disease at Mecca and in the Hedjaz in the autumn of last year. Mohammedan pilgrims make their way from the Mohammedan population, in Western Soudan, across the whole breadth of the African Continent to Mecca, and it is surmised that these pilgrims, returning from the Holy Land of the Moslems at the time when cholera was prevailing there, introduced the disease to the banks of the Niger.—*London Lancet.*

---

AT a séance of the French Academy of Medicine held May 9, Dr. Blanche read a communication discussing whether insanity should be considered a ground for divorce. His negative answer was subscribed to by Magnau and Charcot. It was based on the possibility of recovery. Even in incurable cases, there are phases of remission preceding the time when the disease ultimately became incurable, but in this case, death was not often long delayed.—*Arch. Gén. de Méd.*

INSANITY AND DIVORCE.—A recent English decision (*Med. and Surg. Reporter*) is of some interest in this connection. On the night of the marriage, the wife refused to allow it to be consummated. She was subsequently found, on examination by several alienists, to be a melancholiac, and mentally unfit to enter into a marriage contract. The court decided the marriage was null.

The editor of the *Journal of Nervous and Mental Disease*, commenting on the late resignation of all the members of the post-graduate faculty of the University of the City of New York, believes that a post-graduate school of Medicine will be started, in which the teaching of neurology and psychiatry will hold an important position.

René has made a large number of experiments upon the rate of transmission of nerve force in man. He obtained $\frac{3.4}{100}$ of a second as the approximative duration of a cerebral act; and a rate of transmission of nerve force in a sensory nerve of 28 meters a second.

Paul Bert, following in the steps of M. Béchamp, has, by a series of experiments, discovered that oxidized water arrests fermentation from the presence of living organisms, but is inert in the presence of amorphous ferments (diastase, saliva, pancreatic juice, etc.)

Even the rudest and most empirical ideas of diseases presuppose ideas of the healthy states from which they are deviations; and obviously the diagnosis of diseases can become scientific only as there arises scientific knowledge of organic actions that are undiseased.

The idea that iron is an analeptic has come to an end. Alimentation is more than sufficient to furnish the two or three grams of iron which are contained in the totality of an adult's blood.—Dr. Luton, in *L'Union Médicale.*

